# A multiplex network approach for the analysis of intracranial pressure and heart rate data in traumatic brain injured patients

**DOI:** 10.1007/s41109-017-0050-3

**Published:** 2017-08-30

**Authors:** Giovanna Maria Dimitri, Shruti Agrawal, Adam Young, Joseph Donnelly, Xiuyun Liu, Peter Smielewski, Peter Hutchinson, Marek Czosnyka, Pietro Lió, Christina Haubrich

**Affiliations:** Computer Laboratory, University of Cambridge, Thomson Avenue, Cambridge, UK

**Keywords:** Multiplex time series network, Visibility graph, ICP

## Abstract

**Background:**

We present a multiplex network model for the analysis of Intracranial Pressure (ICP) and Heart Rate (HR) behaviour after severe brain traumatic injuries in pediatric patients. The ICP monitoring is of vital importance for checking life threathening conditions, and understanding the behaviour of these parameters is crucial for a successful intervention of the clinician. Our own observations, exhibit cross-talks interaction events happening between HR and ICP, i.e. transients in which both the ICP and the HR showed an increase of 20% with respect to their baseline value in the window considered. We used a complex event processing methodology, to investigate the relationship between HR and ICP, after traumatic brain injuries (TBI). In particular our goal has been to analyse events of simultaneous increase by HR and ICP (i.e. cross-talks), modelling the two time series as a unique multiplex network system (Lacasa et al., Sci Rep 5:15508-15508, 2014).

**Methods and data:**

We used a complex network approach based on visibility graphs (Lacasa et al., Sci Rep 5:15508-15508, 2014) to model and study the behaviour of our system and to investigate how and if network topological measures can give information on the possible detection of crosstalks events taking place in the system. Each time series was converted as a layer in a multiplex network. We therefore studied the network structure, focusing on the behaviour of the two time series in the cross-talks events windows detected. We used a dataset of 27 TBI pediatric patients, admitted to Addenbrooke’s Hospital, Cambridge, Pediatric Intensive Care Unit (PICU) between August 2012 and December 2014.

**Results:**

Following a preliminary statistical exploration of the two time series of ICP and HR, we analysed the multiplex network proposed, focusing on two standard topological network metrics: the mutual interaction, and the average edge overlap (Lacasa et al., Sci Rep 5:15508-15508, 2014). We compared results obtained for these two indicators, considering windows in which a cross talks event between HR and ICP was detected with windows in which cross talks events were not present. The analysis of such metrics gave us interesting insights on the time series behaviour. More specifically we observed an increase in the value of the mutual interaction in the case of cross talk as compared to non cross talk. This seems to suggest that mutual interaction could be a potentially interesting “marker” for cross talks events.

## Introduction and background

Cerebral blood flow together with cerebrospinal fluid dynamics (CSF) determine the value of the Intracranial Pressure (ICP) (Czosnyka and Pickard 2004), that is the pressure happening inside the brain tissue and the CSF. ICP can be affected and altered due to traumatic brain injury (TBI) and other neurocritical conditions of the central system, that can affect dramatically its behaviour (Czosnyka and Pickard 2004). The ICP monitoring requires the application of an intracranial pressure transducer, and can be continuosly checked in patients affected by severe brain injuries or similar life threatening conditions (Czosnyka and Pickard 2004; Hu et al. 2009). The information contained in the ICP signal is of vital importance to predict critical medical situations such as intracranial hypertension, i.e. ICP peaks. Increase of the ICP can in fact lead in the worst cases to the death of the patient, and the analysis of elements that could possibly signal the presence of such condition, is of vital importance. To the best of our knowledge only a few works concentrate on the identification of a model describing the intracranial system behaviour. For example in (Hu et al. 2007) a hidden state estimation algorithm is used for the estimation of unobserved measurements, such as ICP and cerebral blood flow velocity (CBFV). This is a two steps model, in which parameters of a modified nonlinear intracranial mathematical model are first identified in an offline stage. Subsequently a nonlinear Kalman filter estimator is applied to evaluate unobserved variables, given some measurements such as ICP and cerebral blood flow velocity (CBFV). The relationship of ICP with respect to other monitored parameters is in fact a key aspect to study. An example of this is (Hu et al. 2008). Here the authors present ApEN, an algorithm based on the adaptive calculation of approximate entropy, integrated with a causal coherence analysis that is able to exploit the potential interaction between ICP and R wave intervals (Hu et al. 2008). On the other hand, in (Hu et al. 2007) the authors extract indices of causal coherence and generalized synchronization, considering beat to beat mean intracranial pressure measurements and intervals between consecutive normal sinus heartbeat (ICP and RR intervals). Starting from own visual observations in the dataset described in the following section, we noticed the presence of cross-talks interaction events happening between the HR and ICP time series. We decided therefore to model the phenomenon, via complex event processing methods. Complex network models have been widely applied in many fields, due to the capability of capturing interesting properties of very different type of systems (Albert and Barabási 2002; Newman 2010). Networks analysis in fact can capture irregular systems structures, together with their complex and dynamic evolution, and can be suitable for the analysis of large heterogenous types of systems (Boccaletti et al. 2006). Quite recently the science of complex networks has been applied to time series analysis. An example of construction of complex networks from pseudoperiodic time series is (Zhang and Small 2006). In this paper the authors show how noisy time series correspond to random networks, while chaotic time series exhibit small world and scale free properties (Zhang and Small 2006). An interesting application of such approach was made in terms of comparison between healthy and coronary care patients (Zhang and Small 2006). Therefore the underlying nature of the two time series could be detected by looking at their network representation. Another interesting approach has been proposed by (Marwan et al. 2009). In the paper the authors compute the recurrence matrix of the time series, and use it as the adjacency matrix of the complex network. Then they analyse such network, using standard network metrics. A further important methodology that links time series and complex networks is the, so called, visibility graph approach (Lacasa et al. 2014). In particular visibility graphs are a family of methods that were used extensively in literature in recent years (Lacasa et al. 2008). Applications are in different areas from climate dynamics (Donges et al. 2009), to the analysis of the gold price time series (Long 2013), to the detection of sequential motifs in visibility graphs (Iacovacci and Lacasa 2016). An extensive review of the applications of such methodology is done in (Nuñez et al. 2012). More recently this approach has been extended to the case of multivariate time series, as proposed in (Lacasa et al. 2014). This allows to map a multivariate time series into a multi-layer network (Bianconi 2013; Kivelä et al. 2014) in the so called multiplex visibility graph (Lacasa et al. 2014) (see Methods for details). From such model, using the metrics of complex network theory, interesting insights and new information on the behaviour of the multivariate time series can in fact be detected. Therefore, starting from the visual observation that HR and ICP present peaks at similar points in time, we first performed an explorative statistical analysis on the correlation between HR and ICP time series. We then implemented a naive sliding window approach to the two time series, to detect cross talks events between the two parameters. The two time series were then modelled as a multiplex visibility graph network. Multilayer graph metrics were then obtained to investigate and analyse the behaviour of HR-ICP relationship during cross talks events.

## Dataset

Data were collected prospectively from 27 pediatric TBI patients admitted to Addenbrooke’s Hospital, Cambridge, Pediatric Intensive Care Unit (PICU), between August 2012 and December 2014. TBI patients with a clinical need for ICP monitoring were included for analysis. The insertion of an intracranial monitoring device is a standard in clinical practice and as such did not require ethical approval. Data are routinely collected for clinical purposes and guide the management of patients. The analysis of data within this study for the purposes of service evaluation, was approved by the Cambridge University Hospital NHS Trust, Audit and Service Evaluation Department (Ref:2143) and did not require ethical approval or patient consent. Several different parameters were collected such as ABP mean arterial pressure (mmHg), HR heart (Hz) an ICP intracranial pressure (mmHg). The data sampling rate was 200 Hz.

## Methods

We first performed an exploratorive analysis based on standard time series techniques. For an early intuition on the dynamics of the system, we decided to use the recurrence plots (RP) (Eckmann et al. 1987; Marwan et al. 2007) to identify the possibility of similar behaviour happening between the HR and the ICP of each patient. RP is a statistical technique used for non linear data. Data are visualized through a graph in a square matrix (columns and rows represent a pair of time points), and the elements are representation of the times at which a state of the dynamical system recurred (Recurrence Plots 2017).

### A sliding window approach for cross-talks detection

We implemented a naive sliding window approach for the detection of cross talks events in the time series (Dimitri et al. 2017). The goal was to identify the presence of episodes, where both ICP and HR increased by 20% with respect to the minimum value in a given window of the time series. The algorithm implemented works as follows:

1. Consider two time series *X*=*x*_1_,*x*_2_,*x*_3_,...,*x*_*N*_ and *Y*=*y*_1_,*y*_2_,...*y*_*N*_
2. Consider a window *W* of length *L* that slides across the whole length of the time series simultaneously
3. If in both time series the maximum value in the *i*−*th* time window considered is at least a 20% higher than the minimum value in this time window, and if after the maximum value there is a decrease of at least 20% then a cross talk event is detected.

Such approach gave us the possibility to identify the presence of a number of crosstalks events happening in the two time series as we summarize in Table 1. The number of such events found per patient has a high interval of variation ranging from 184 to 0. This can depend on many different factors such as the seriousness of the patient condition. The threshold of 20%, as well as the length of 10 minutes for the time window, were selected according to clinical and medical observations, but different windows length for observation can be studied according to clinical reasons. In particular 10 minutes is a reasonable length for the possibility of a timely intervention by the clinician. The code written, in R and available upon requests from the authors, allows to try and evaluate differente thresholds and different windows lengths.

**Table 1:** Number of cross talks events for each patient detected by the naive sliding window approach

P1	P2	P3	P4	P5	P6	P7	P8	P9
15	35	66	20	1	23	29	43	59
P10	P11	P12	P13	P14	P15	P16	P17	P18
69	22	142	31	29	7	36	1	0
P19	P20	P21	P22	P23	P24	P25	P26	P27
1	20	184	57	2	15	0	16	18

### Multilayer networks

The area of multilayer networks has seen an increasing interest and applicability in many different fields in recent years. A multilayer network can be defined as *M*=(*G,C*) where *G* is a set of graphs and *C* is the interconnection between them (Boccaletti et al. 2014). Several important metrics and descriptors of single layer networks have been generalized to the case of multilayers. For example in (De Domenico et al. 2013) the authors present a tensorial framework to study multi-layer networks and present many different topological metrics, generalized for the case of a multilayer approach. Other important works concentrate on the generalization of concepts, such as community detection, to the multilayer case. An example is (Mucha et al. 2010). Here the authors focus on the extension of the community detection approach to a multilayer network in a time dependent and multiscale environment. In (Cozzo et al. 2015) for example they generalize the concept of clustering coefficient for multilayer networks, showing drawbacks and difficulties of the generalization procedure from single layer to multi layer networks. In (De Domenico et al. 2016) the authors provide a deep and extended description of processes on multilayer networks, highlighting some of the physical phenomena that are related to spreading processes. A specific example of a multilayer network is the multiplex network in which each layer has the same nodes. Therefore the interlayer connection happen between each node and the correspondent one in the other layers. Structural measures for multiplex networks have been formalized as (Battiston et al. 2014) shows.

### The multiplex horizontal visibility graph

To map the time series into a graph, we adopted the Visibility Graph approach (Lacasa et al. 2014; Lacasa et al. 2012). Two variants of the visibility graph mapping exist: the Natural Visibility Graph (NVG) and the Horizontal Visibility Graph (HVG). The former is based on the following criterium: each node in the graph corresponds to a time stamp and two nodes share an edge if the two time stamps *can see* each other (Lacasa et al. 2008). This means that given a time series of *N* data points and given two points *z* and *t*, with their corresponding values *y*_*z*_ and *y*_*t*_, these two will be connected by an edge, if for a given value *y*_*l*_ between *y*_*z*_ and *y*_*t*_ the following holds (Lacasa et al. 2008):

1 $$ y_{l}<y_{t}+(y_{z}-y_{t})\frac{t-l}{t-z}  $$

The second criterium, the so called Horizontal Visibility Criterion, works as follows: two nodes *t* and *z* share an edge connection in the horizontal visibility graph if given any other time values *x*_*l*_ the following holds:

2 $$ x_{l}<inf(x_{t},x_{z}), \forall l:t<l<z  $$

The latter version of the visibility graph is computationally more tractable and presents interesting outplanar properties that makes it easier to interact and deal with. Complexity of both algorithms is low, with a *O*(*NlogN*) for both NVG and HVG. We decided to use the HVG in our experiments. This is due to the fact that HVG have been shown to work well with short correlation and short-scale visibility correlation, as they typically present an exponentially decaying degree distribution (Sannino et al. 2017). Since we were interested in analysisng the behaviour of the multivariate time series system formed by ICP and HR, we adopted the multiplex visibility graph approach of (Lacasa et al. 2014). Suppose in fact to have *M* time series. Then following the visibility graph approach, each time series can be mapped as a layer in the multilayer representation, and since every graph in each layer presents the same set of nodes (the temporal time stamp *t*), this is the so called multiplex graph (Battiston et al. 2014; Boccaletti et al. 2014). In our modelling we proceeded as follows:

1. We used the naive sliding window approach to obtain significant non overlapping windows, in which a cross talk event was detected based on the definition previously stated.
2. We mapped each time series window in which a cross talk was detected into a graph following the HVG approach
3. We performed graphs and network statistics as described in the results section

For the complete reference on the mathematical model please refer to: (Lacasa et al. 2008; Lacasa et al. 2012; Luque et al. 2009). We used two metrics for the evaluation of the multilayer graph. The first indicator is the average edge overlap. Such metric is defined in (Battiston et al. 2014; Bianconi 2013; De Domenico et al. 2013) as:

3 $$ \langle O \rangle=\frac{1}{k}\sum\limits_{i,j}o_{ij}  $$

where $o_{ij}=\frac {1}{M}\sum \limits _{\alpha }\alpha _{ij}^{[\alpha ]}$. The average edge overlap is a way to quantify the coherence of the overall graph and the higher it is, the higher the coherence of the graph layers. The second metric is the interlayer mutual information: this is defined as (Lacasa et al. 2014):

4 $$ I_{\alpha,\beta}=\sum\limits_{k^{[\alpha]}k^{[\beta]}}P(k^{[\alpha]},k^{[\beta]})log\frac{P(k^{[\alpha]},k^{[\beta]})}{P(k^{\alpha})P(k^{\beta})}  $$

and in this case *P*(*k* ^[*α*]^,*k* ^[*β*]^) is the joint probability of having a node with degree *k* ^[*α*]^ at layer *α* and of degree *k*[*β*] at layer *β*. Such measure is in part limited to the fact that only the degree distribution of the network is considered. More sophisticated and complete measures exist, as shown in (De Domenico et al. 2015).

## Results and discussion

**Fig. 1:**
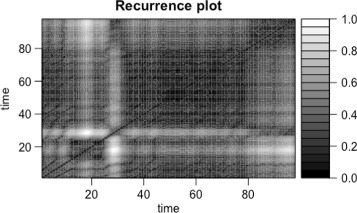
Recurrence Plots. The figure shows the recurrence plots of the ICP for a patient.The parameters used for the recurrence plots were (d=2 and m=3, where m is the embedding dimension and d is the delay)

**Fig. 2:**
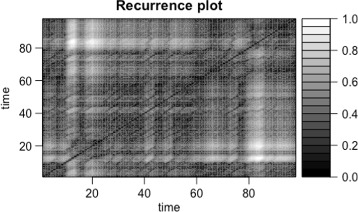
Recurrence Plots. The figure shows the recurrence plots of the HR time series for a patient.The parameters used for the recurrence plots were (d=2 and m=3, where m is the embedding dimension and d is the delay)

We present here the results of our analysis. In Figs. 1 and 2 we show two recurrence plots, respectively, for the HR and ICP time series of the patients. In this figure the black dots represent recurrent points in the system and the lines parallel to the diagonal, show the determinism of the two time series considered. Moreover the similarity in the recurrence plots suggests the presence of an interaction between the two time series (Dimitri et al. 2017).

**Fig. 3:**
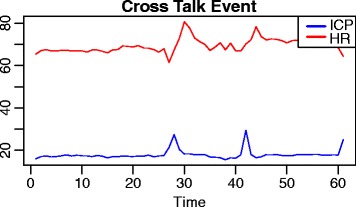
Cross Talk Event Event Plot. The figure shows the plot of HR and ICP time series, in a time window where a cross talk event is detected

**Fig. 4:**
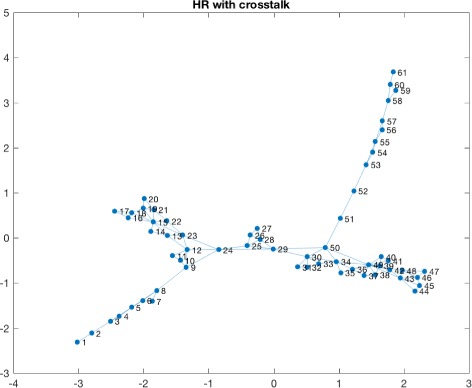
HR graph with cross talk. The figure shows the HR horizontal visibility graph corresponding to the time window in which a cross talk is detected and shown in Fig. 3

**Fig. 5:**
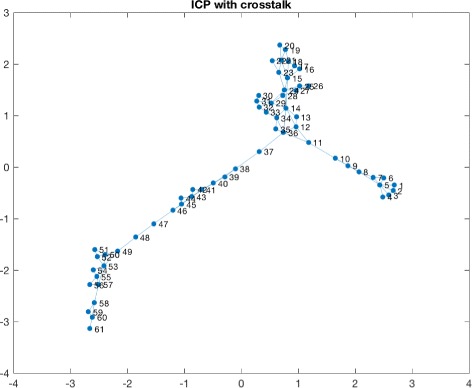
ICP graph with cross talk. The figure shows the ICP horizontal visibility graph corresponding to the time window in which a cross talk is detected and shown in Fig. 3

**Fig. 6:**
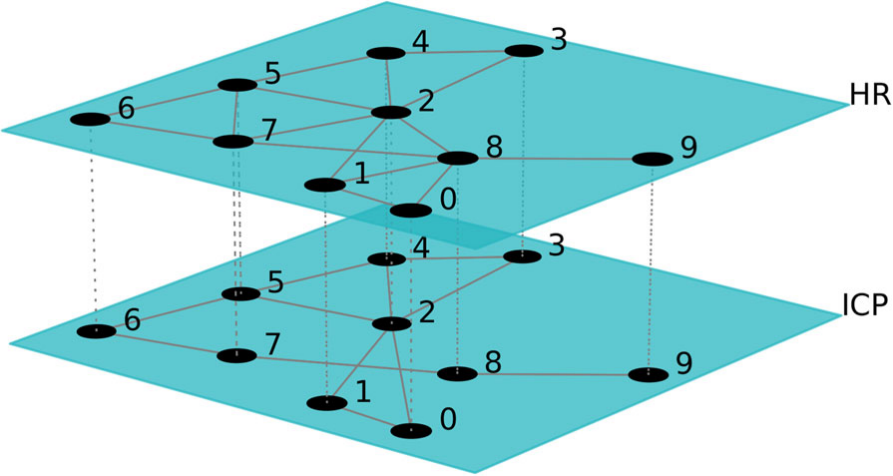
Visualization of the multiplex visibility graph for the ICP and HR with a cross talk event: Multiplex Visibility graph of ICP and HR in the time window with a cross talk event detected (Mikko 2017)

**Fig. 7:**
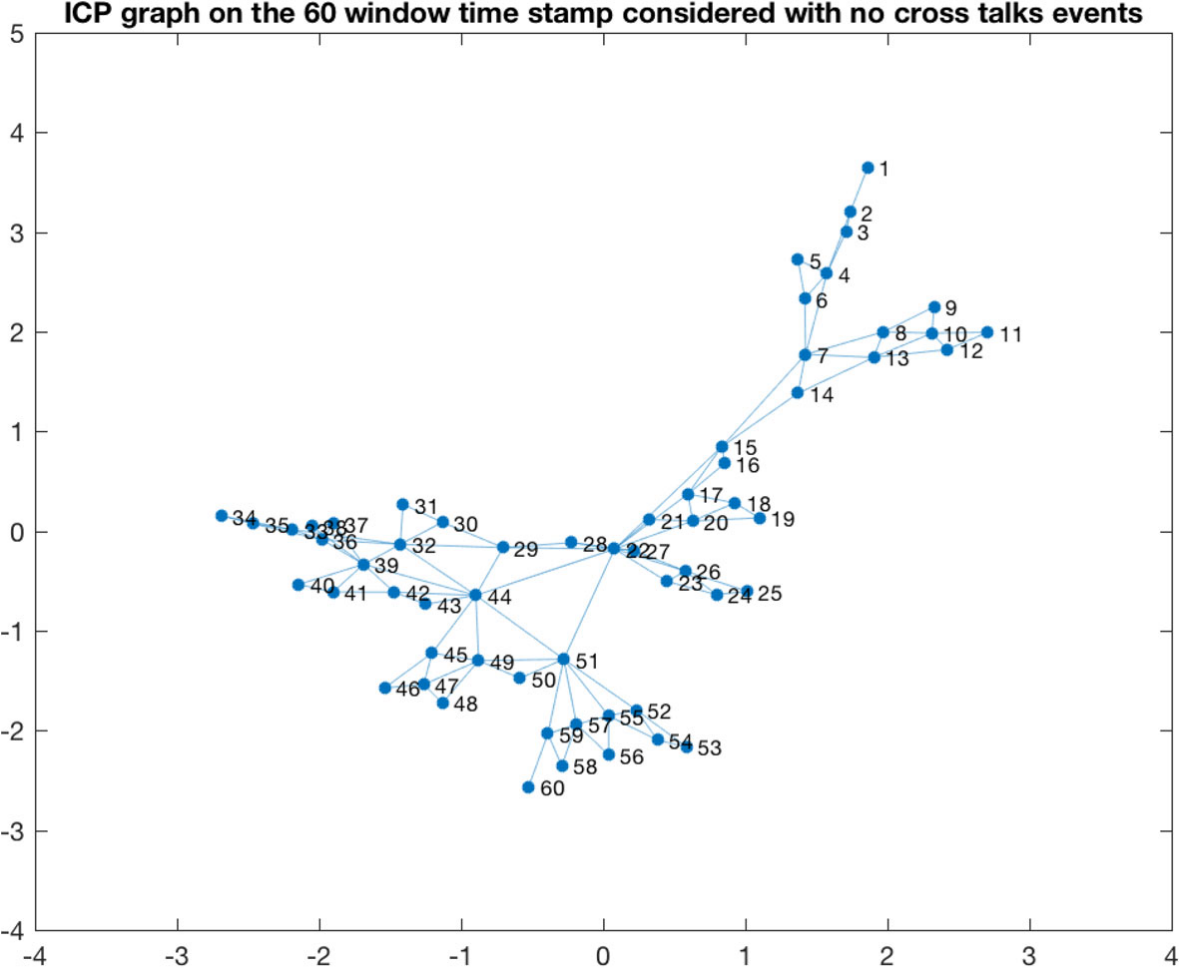
ICP graph with no cross talk. The figure shows the ICP horizontal visibility graph corresponding to the time window in which no cross talks events are detected

**Fig. 8:**
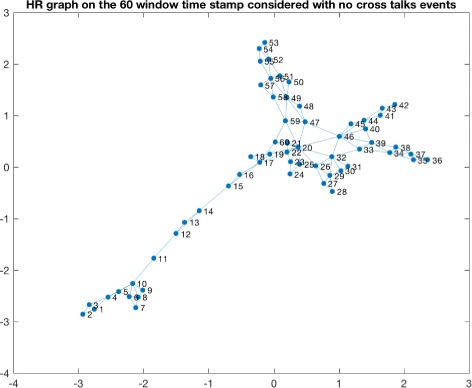
HR graph with no cross talk. The figure shows the HR horizontal visibility graph corresponding to the time window in which no cross talks events are detected

Consequently we applied a naive sliding window approach to detect windows in which cross talks events were taking place. This led to the identification of various candidate windows. We observed a high variability in terms of the number of events detected per patients and this can be due to many different reasons, starting from the severity of the trauma and other biological meanings. The time window considered for detecting the presence of a cross talk event is 10 minutes of observations. The number of cross talks events detected for each patient is summarized in Table 1. Figure 3 shows a window in which a cross talk event is identified for one of the patients. Once obtained the window, we proceeded to the transformation of that time series window into a HVG. As an example in Fig. 4 and in Fig. 5 we show the horizontal visibility graphs obtained from the cross-talk event from Fig. 3 and in Fig. 6 we show the multiplex structure of the two graphs obtained for the time window considered. In such graph we obtain the following results for the average edge overlap and the mutual interaction information parameters of the multiplex network: *ω*=0.7920, *Interlayer Mutual Information* =0.7285. As an example in Fig. 7 and in Fig. 8 we show the graphs of the ICP and HR where no cross talks events were detected. In this case we obtain: *ω*=0.6029, *Interlayer Mutual Information* =0.7035. For a summary measure regarding the *ω* and the *Interlayer Mutual Information* we proceeded as follows: for each patient we considered 10 windows in which a cross talk was detected and 10 windows in which no cross-talk was present. Then we computed the average value of the *ω* average edge overlap, the mutual information for the 10 windows with cross talks and the 10 windows without cross talks. The results of such analysis are shown in Table 2. We chose 10 because it seemed a reasonable number to take, given the high variability of cross talks events across patients. For this reason we discarded only a few patients. Considering the table, we can see how there seems to be a trend in the way the *ω* and the Mutual Interaction behaves with respect to Cross Talks and non Cross Talks events. In particular there seems like the *ω* value is more stable between the two cases. On the other hand, the Mutual Interaction, has a more clear trend of increasing on average when a cross talk event is detected as compared to when there is no cross talk. This is in fact shown in the last row of the Table 2 where the average values of each column are presented. This would suggest that when a cross talk event takes place, the two time series are more similar than when no cross talks events are detected. Such preliminary findings suggest therefore how the network topology metrics considered so far, could be of extreme importance for a further analysis of the system. In fact there could be the possibility of using a network modelling in order to extract topological features that could be used for the prediction of crosstalks events happening in the case of brain traumatic patients.

**Table 2:** Average value of the average edge overlat and mutual interaction for cross talks and non cross talks events windows

Patient	*ω* CT	MI CT	*ω* nonCT	MI nonCT
1	0.7535	0.5690	0.7513	0.4721
2	0.7334	0.4913	0.7306	0.4633
3	0.7444	0.5782	0.7388	0.4155
4	0.7424	0.6424	0.7298	0.5522
6	0.7505	0.5752	0.7544	0.6037
7	0.7715	0.4113	0.7630	0.2915
8	0.7431	0.5370	0.7277	0.5831
9	0.7382	0.6013	0.7552	0.6202
10	0.7301	0.5516	0.7399	0.4434
11	0.7473	0.5233	0.7633	0.3407
12	0.7346	0.6232	0.7243	0.5017
13	0.7635	0.4901	0.7622	0.3662
14	0.7420	0.6017	0.7540	0.6219
16	0.7611	0.5504	0.7495	0.6464
20	0.7260	0.5716	0.7321	0.5587
21	0.7283	0.4647	0.7272	0.4721
22	0.7191	0.5154	0.7545	0.5996
24	0.7520	0.6271	0.7654	0.4976
26	0.7565	0.4729	0.7306	0.4367
27	0.7818	0.7818	0.7764	0.3088
Average values	0.7460	0.5590	0.7465	0.4898

Each row is a patient. Every value presented is averaged over 10 cross talk windows. We discarded patient 5,15,18,19,23,25 who had less than 10 cross talks detected. CT and non CT stands for cross-talk or non cross-talk event

## Conclusion and future directions

We present here a multiplex network model for the analysis of multivariate time series. In particular we analysed the behaviour of the intracranial pressure (ICP) and the heart rate (HR) in a cohort of 27 pediatric brain traumatic patients. We first applied some basic statistical techniques, such as recurrence plot, to study the behaviour of the two time series. Afterwards we applied a naive sliding window approach to detect the presence of cross-talks and non cross-talks events. We then modelled our system using the multivariate time series horizontal visibility graph approach as described in (Lacasa et al. 2014). In particular we analysed the behaviour of the multivariate system considering two multilayer network metrics: the average edge overlap and the interlayer mutual correlation. We decided to use these two measures as classical indicators adopted in the literature for a first endeveour to analyse the system. We evaluated the average trend of these two metrics on 10 cross-talks and non cross-talks events for each patient. Findings suggest that while the average edge overlap seems to have a more stable behaviour between the two situations, the mutual interaction on the other hand shows a more clear trend. In particular the average value increases when cross talks events are detected, meaning that the two time series behaves more similarly in this case. Future directions of research includes the integration of further parameters that are monitored in this cohort of patients, and that could help in the analysis and understanding of the cross talks behaviour. We therefore plan to extend our multiplex model, also considering further multiplex network properties and measures in the analysis and integrating the biological knowledge regarding the system into its network representation.
